# Enhanced detection of atrial fibrillation in single-lead electrocardiograms using a Cloud-based artificial intelligence platform

**DOI:** 10.1016/j.hrthm.2024.12.048

**Published:** 2025-07

**Authors:** François De Guio, Michiel Rienstra, José María Lillo-Castellano, Raquel Toribio-Fernández, Carlos Lizcano, Daniel Corrochano-Diego, David Jimenez-Virumbrales, Manuel Marina-Breysse

**Affiliations:** 1 IDOVEN Research, Madrid, Spain; 2 Department of Cardiology, University of Groningen, University Medical Center Groningen, Groningen, The Netherlands; 3 Centro Nacional de Investigaciones Cardiovasculares (CNIC), Myocardial Pathophysiology Area, Madrid, Spain; 4 Hospital Universitario del Henares, Spain; 5 Centro de Investigación Biomédica en Red. Enfermedades Cardiovasculares (CIBERCV), Madrid, Spain

**Keywords:** Artificial intelligence, Atrial fibrillation, ECG analysis, Deep learning, Remote screening

## Abstract

**Background:**

Although smartphone-based devices have been developed to record 1-lead electrocardiogram (ECG), existing solutions for automatic detection of atrial fibrillation (AF) often has poor positive predictive value.

**Objective:**

This study aimed to validate a Cloud-based deep-learning platform for automatic AF detection in a large cohort of patients using 1-lead ECG records.

**Methods:**

We analyzed 8528 patients with 30-second ECG records from a single-lead handheld ECG device. Ground truth for AF presence was established through a benchmark algorithm and expert manual labeling. The Willem Artificial Intelligence (AI) platform, not trained on these ECGs, was used for automatic arrhythmia detection, including AF. A rules-based algorithm was also used for comparison. An expert cardiology committee reviewed false positives and negatives, and performance metrics were computed.

**Results:**

The AI platform achieved an accuracy of 96.1% (initial labels) and 96.4% (expert review), with sensitivities of 83.3% and 84.2%, and specificities of 97.3% and 97.6%, respectively. The positive predictive value was 75.2% and 78.0%, and the negative predictive value was 98.4%. Performance of the AI platform largely exceeded the performance of the rules-based algorithm for all metrics. The AI also detected other arrhythmias, such as premature ventricular complexes, premature atrial complexes along with 1-degree atrioventricular blocks.

**Conclusion:**

The result of this external validation indicates that the AI platform can match cardiologist-level accuracy in AF detection from 1-lead ECGs. Such tools are promising for AF screening and have the potential to improve accuracy in noncardiology expert health care professional interpretation and trigger further tests for effective patient management.

## Introduction

Atrial fibrillation (AF) is the most common sustained cardiac arrhythmia, occurring in 1% to 2% of the general population and 8% of individuals aged 65 years and older^.^^1^^,^^2^ Patients with AF have 50% to 90% higher mortality rates, presenting higher risk of death, stroke, dementia, heart failure, and other cardiovascular diseases according to the Framingham Heart Study.^2^ The global prevalence of AF is approximately 60 million cases, and the lifetime risk of AF is approximately 33%.^3^

Detection of AF remains challenging, as it may be episodic and may be associated with unspecific or no clinical symptoms. Therefore, AF is frequently subclinical and elusive, making diagnosis and treatment difficult. Nevertheless, AF screening and detection is essential to improve clinical outcomes through early interventions.

Internet of things (IoT) and artificial intelligence (AI) technologies have driven the development of wearable electrocardiograph (ECG) devices used for health monitoring, which are an effective means of AF detection. AI has enabled wearable technology to aid in the passive detection of cardiac arrhythmias and enhanced the diagnostic power of ECG in different environments and different patients' journeys.^4^^,^^5^

The KardiaMobile (AliveCor, Mountain View, CA) handheld device is a smartphone-based 1-lead ECG recorder. It is a portable device with 2 electrodes being able to write a single bipolar ECG lead using a smartphone app. Its ease of use allows it to be used for large-scale AF screening, especially outside of traditional health care settings.^6^ Nevertheless, recent studies highlighted that automated 1-lead ECG interpretation, although showing reasonable positive predictive value (PPV) was not sufficiently accurate to replace manual overread or confirmatory testing. Indeed, only 52% of tracings interpreted as AF by the automated proprietary algorithm were confirmed to show AF upon cardiologist review.^6^

The purpose of the current study is to evaluate the performance of Willem AI platform (Idoven, Madrid, Spain), a device-neutral deep-learning based platform, to detect AF from single-lead ECG records acquired with the KardiaMobile handheld device. Importantly, the goal is to evaluate the classification performance of the AI platform in an external dataset: specifically, a large dataset that was not employed during the training of the deep-learning algorithm. Our hypothesis is that such AI platform could reach better accuracy and notably better PPV than the reported 52% to detect AF from 1-lead ECG,^6^ which means it has the potential to enhance the capability to identify clinically relevant diagnoses and improve the user experience for both health care professionals and patients using these medical-grade tools for management of cardiovascular disease. The AI platform was also compared with a benchmark non-AI rules-based algorithm for AF detection.

## Methods

### Data source

All 1-lead ECG records originate from the 2017 PhysioNet/Computing in Cardiology (CinC) Challenge.^7^ ECG recordings were collected using the KardiaMobile device. Each recording was taken by an individual who had purchased 1 of 3 generations of KardiaMobile single-channel ECG device and, in theory, held each of the 2 electrodes in each hand, creating a lead I (LA-RA) equivalent ECG. After some basic checks for signal quality, the device recorded for an average of 30 seconds. The hardware then transmitted the data to a smartphone or tablet acoustically into the microphone (over the air, not through a wire) using a 19 kHz carrier frequency and a 200 Hz/mV modulation index. The data were digitized in real time at 44.1 kHz and 24-bit resolution using software demodulation. Finally, the data were stored as 300 Hz, 16-bit files with a bandwidth 0.5-40 Hz and a ± 5 mV dynamic range. The data were then converted into WFDB-compliant Matlab V4 files (each including a .mat file containing the ECG and a .hea file containing the waveform information) and split into training and test data sets. The challenge training set contains 8528 recordings lasting from 9 seconds to 61 seconds, whereas the challenge test set contains 3658 recordings of similar lengths and was not available. Then, only the challenge training set was considered in the current study. It is important to note that the Willem AI platform has already been trained and validated using other datasets from more than 520,000 patients (see ***AI platform*** section). Therefore, the entire current dataset of 8528 recordings was used solely for the external validation of the algorithm to detect AF, meaning the AI platform was not trained with any data included in this dataset.

### Challenge expert labeling

Four versions (V0, V1, V2, and V3) of the labels have been defined from this dataset for the Challenge purpose. The V3 version was chosen here as the ground truth to compare with the AI platform output given it was the most advanced version. The labeling process involved both a benchmark algorithm and manual labeling by cardiology experts for cases in which consensus among the top algorithms was not achieved.^7^

A total of 5076 ECG records were labeled as "Normal" (59.5%), 758 records as "AF" (8.9%), 2415 records as "Other" (28.3%), and 279 records as "Noisy" (3.3%).

### AI platform

The Willem AI platform (Idoven, Madrid, Spain) is a deep learning-based CE-mark software as a medical device (SaMD) to analyze ECGs from a very large variety of ECG recording devices: 12-leads ECGs, Holters, ambulatory cardiac monitors such as patches and textiles, insertable cardiac monitors (ICMs), 1-lead and 2-leads ECG wearables, and handheld ECG devices, among others.

The Willem AI platform’s deep-learning algorithms have been trained to detect up to 23 types of cardiac patterns and arrhythmias and have been validated both internally and externally using a large database, which contains ECG records from more than 520,000 patients. At present, this database includes proprietary but also public short- and long-term ECG records extracted from registries such as the PTB-XL database,^8^ The Georgia 12-leads ECG challenge,^9^ the European Society of Cardiology database,^10^ the American Heart Association database,^9^ and the MIT-BIH Arrhythmia database.^11^ More in depth, selected record subsets from 323,794 patients were independently used for training or internal validation. In addition, 13,240 patients’ records coming from the EC57 standard and 2 external databases (Georgia^9^ and Stanford) were used for external validation. Further information about the AI deep learning algorithms development has been previously explained in Quartieri et al.^12^

This AI platform has been notably used for efficient ICM episodes triage reducing false positive (FP) detections by 98%,^13^ extending the number of cardiac patterns detected from 4 to 25, and reducing the ECG analysis time from 11 minutes to 6 seconds.^12^ It was also recently used to identify cardiac patterns from Holter’s records as a tool to standardize a remote heart-screening program.^14^

For each ECG record, the AI platform automatically outputs a list of detected and undetected cardiac patterns along with interval measurements. In this study, only AF pattern classification could be evaluated against experts, given that other cardiac patterns were not annotated in the Challenge.

### External cardiology expert committee labeling

Given that not all ECG labels from the Challenge training dataset were meticulously evaluated by experts, additional cardiology experts were engaged to verify AF labels in cases with which there was a discrepancy between expert labeling and the AI output (also in some true positive [TP] and true negative [TN] cases for illustration purpose). Subsequently, an additional evaluation of the AI platform was conducted against this newly verified labeling, supplementing the initial evaluation against the Challenge expert labeling.

### Rules-based algorithm

The rules-based algorithm is based on the analysis of heart beat variability from interbeat intervals obtained by a wavelet-based detector as described in Park et al.^15^ As the final classifier module is proprietary and relies on a support vector machine trained by that research team, a thresholding-based final classifier was applied as the final module characterizing the presence or absence of AF.

### Performance metrics

Accuracy, sensitivity, specificity, PPV, negative predictive value (NPV), and F1-score for AF classification were calculated considering the AI platform’s performance vs the Challenge expert labeling or the external cardiology expert committee labeling.

Based on the number of TP, TN, false positive (FP) and false negative (FN) cases, performance metrics were computed as follows, using R statistical software (R Core Team [2024]. R: A Language and Environment for Statistical Computing, R Foundation for Statistical Computing, Vienna, Austria):

$$Accuracy=\frac{TP+TN}{TP+TN+FP+FN},Sensitivity=\frac{TP}{TP+FN},Specificity=\frac{TN}{TN+FP},PPV=\frac{TP}{TP+FP},NPV=\frac{TN}{TN+FN},F1-score=\frac{2TP}{2TP+FP+FN}$$

Performance of the AI platform was also compared with the rules-based algorithm considering external cardiology expert committee labeling as ground truth. Metrics differences between the 2 methods were assessed using a McNemar test. A *P* value < .01 was considered to be statistically significant.

For other cardiac patterns detected by the AI, prevalence is reported but no performance metrics are shown because of the absence of ground truth.

## Results

### AI performance in detection of AF

For a total number of 8528 records analyzed, the AI platform performance metrics were calculated in comparison with the Challenge labels but also in comparison with the external cardiologists’ labels for detection of AF. These are shown in Table 1.

**Table 1:** AI platform AF classification performance

Performance metrics	Willem AI vs ground truth (Challenge labeling)	Willem AI vs ground truth (external labeling)
Number of records	8528	8528
Accuracy (%)	96.1	96.4
Sensitivity (%)	83.3	84.2
Specificity (%)	97.3	97.6
PPV (%)	75.2	78.0
NPV (%)	98.4	98.4
F1 score (%)	79.0	80.9

PPV = positive predictive value; NPV = negative predictive value.

PPV was determined to be 78.0%, indicating that 78% of 1-lead ECG records interpreted as AF by the AI platform were confirmed to show AF upon review by cardiologists. NPV of 98.4% shows the good performance of the AI to triage the absence of AF (reducing the burden of nonclinically relevant events).

**Figure 1:**
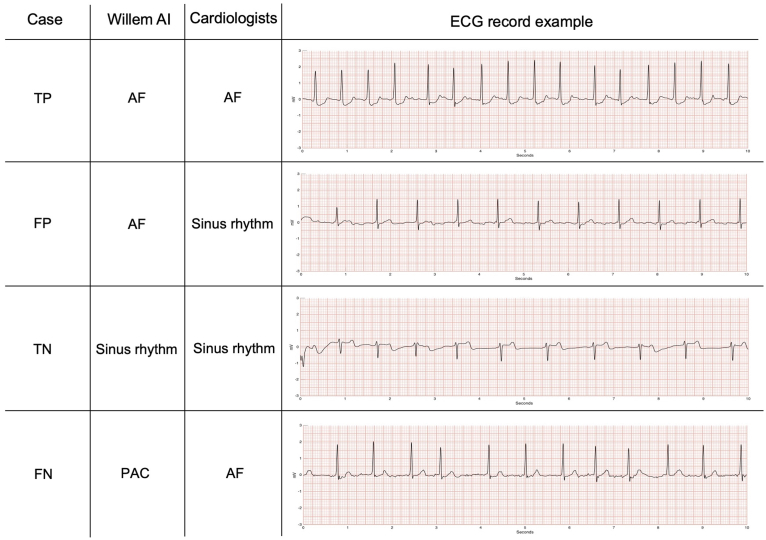
Comparative sample of ECGs interpreted by AI vs cardiologists. The figure shows some ECG records examples that were correctly (true positive and true negative) or wrongly (false positive and false negative) interpreted by the AI Platform in comparison to the cardiologist's diagnosis. True positive (TP) = patient presenting AF according to the AI and cardiologists. False positive (FP) = patient presenting AF according to the AI but not according to cardiologists. True negative (TN) = patient not presenting AF according to the AI and cardiologists. False negative (FN) = patient not presenting AF according to the AI but presenting AF according to cardiologists. These 10-second length ECG examples come from different patient recordings, with a total length of 30 seconds. AF = atrial fibrillation; AI= artificial intelligence; ECG = electrocardiogram; PAC = premature atrial complex.

**Figure 2:**
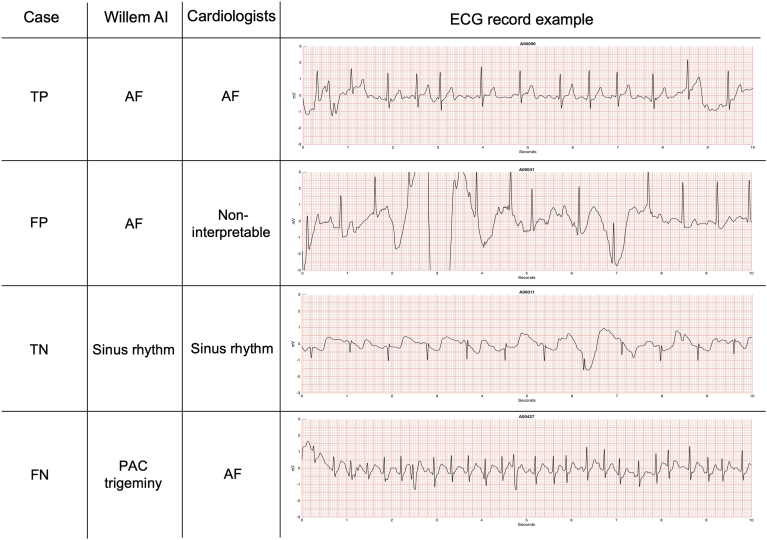
Comparative sample of ECGs with artefacts interpreted by AI vs cardiologists. The figure shows some ECG records examples presenting significant artefacts during the acquisition that were correctly (true positive and true negative) or wrongly (false positive and false negative) interpreted by the AI Platform in comparison to the cardiologist's diagnosis. True positive (TP) = patient presenting AF according to the AI and cardiologists. False positive (FP) = patient presenting AF according to the AI but not according to cardiologists. True negative (TN) = patient not presenting AF according to the AI and cardiologists. False negative (FN) = patient not presenting AF according to the AI but presenting AF according to cardiologists. These 10-second length ECGs examples come from different patient recordings with a total length of 30 seconds. Abbreviations as in Figure 1.

The AI performance to detect AF is illustrated in Figure 1 and Figure 2. Figure 1 shows some cases with accurate classification (TP and TN) as well as a FP and a FN case. The FP ECG record was classified as AF by the AI probably because of the waveform baseline oscillation, which does not allow P waves to be clearly distinguished. Regarding the FN case, the AI misinterpretation might be because of the RR intervals regularity and the precocity of some beats that could be interpreted as premature atrial complexes (PACs).

Figure 2 displays the AI platform classification results in suboptimal conditions such as ECG records with artefacts. Even in the presence of artefacts, the AI accurately detected the presence or absence of AF. The FP example shows that the large number of artefacts and PACs prevented the AI from correctly distinguishing the beats' regularity. The wrong interpretation by the AI in the FN case might be caused by the high rates and aberrant conduction observed in some beats of this AF irregular cardiac rhythm. These beats were considered as PAC trigeminy by the AI as result of a plausible sinus tachycardia promoted by a stress condition.

### AI platform vs rules-based algorithm

**Figure 3:**
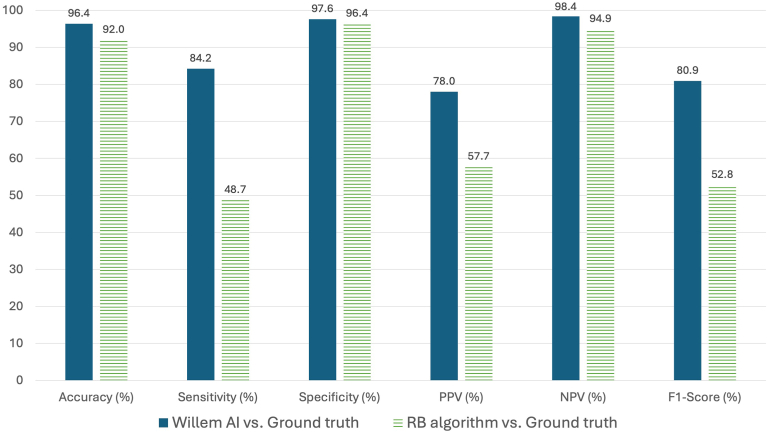
Comparison of performance metrics between AI platform vs rules-based algorithm for detection of atrial fibrillation. This figure shows that the performance of the AI platform for detection of AF largely exceeded the performance of the rules-based algorithm for all metrics and especially a difference in sensitivity of 36%, a difference in PPV of 20% and a difference in F1 score of 28%. All differences between the 2 methods were statistically significant (*P* < .01). PPV = positive predictive value; NPV = negative predictive value; RB = rules-based. Other abbreviations as in Figure 1.

As shown in Figure 3, the performance of the AI platform for AF detection largely exceeded the performance of the rules-based algorithm. The difference between boths methods was found statistically significant for all metrics (*P* < .01). In particular the AI platform yielded much higher sensitivity (84.2% vs 48.7%, *P* < .01), higher PPV (78.0% vs 57.7%, *P* < .01) and higher F1 score (80.9% vs 52.8%, *P* < .01).

### Detection of other cardiac patterns

In addition to the detection of AF, which is the focus of the current study, the AI platform identified other cardiac arrhythmias. Although expert labels have not been assessed in this database for other cardiac patterns, Table 2 reports the list of cardiac patterns detected by the AI in more than 1% of cases.

**Table 2:** Additional cardiac patterns detected by the AI platform (prevalence >1%)

Cardiac pattern	Prevalence in the cohort (N, %)
PVC	939, 11.0 %
PAC	601, 7.1 %
1^st^-degree AV block	371; 4.4 %
PAC couplet	233; 2.7 %
PAC trigeminy	192; 2.3 %
PAC bigeminy	191, 2.2 %
PAC quadrigeminy	158, 1.9 %
PVC bigeminy	153, 1.8 %
Sinus tachycardia	136; 1.6 %
PVC quadrigeminy	119, 1.4 %
PVC trigeminy	119, 1.4 %

AV = atrioventricular; PAC = premature atrial complex; PVC = premature ventricular complex.

## Discussion

In a large dataset of 8528 patients with 1-lead ECG record, 1 per individual, the AI platform reached 96.1% global accuracy, with a 83.3% sensitivity and a 97.3% specificity for detection of AF considering initial labels of the challenge as ground truth. When carefully examining cases in which there was a mismatch between initial label and AI platform’s outcome, and after correcting inappropriate labels based on expert cardiologist committee review, global accuracy was 96.4% with a 84.2% sensitivity and a 97.6% specificity. These results demonstrate that such a deep-learning platform, although not trained on similar ECG data from this hardware device, could effectively detect AF from 1-lead ECG signal from KardiaMobile ECG devices.

Although extensive literature suggests that AI techniques may overcome more historical rules-based algorithms for AF detection, the current results confirm this statement by providing quantified comparative results from a large number of records. In particular, the improvement of sensitivity is major and of great importance in any screening program.

Reported PPV of the AI platform from the current study (75.2% using initial labels, 78.0% with additional expert review) is largely superior to the 52% PPV reported from the VITAL-AF (Screening for Atrial Fibrillation in Older Adults at Primary Care Visits) trial in which 412 records out of 797 records defined as possible AF were correctly identified by the KardiaMobile’s automated interpretation compared with cardiologist reference.^6^ These results have been obtained in a large cohort of 14,230 individuals with 31,376 ECG tracings acquired in a primary care setting. Another recent study assessed the feasibility of screening AF using the KardiaMobile 1-lead device in the Netherlands. From a total of 2168 participants, and including the noninterpretable outcomes, this algorithm showed a NPV and PPV for detecting AF of 99.2% and 60.0%, respectively, and a sensitivity and a specificity of 70.9% and 98.8%.^16^ These metrics are in line with the previously cited study, which are inferior to the current study results.

Hannun et al^17^ also evaluated a deep neural network to classify rhythm classes using single-lead ECGs. Interestingly, they reported an average F1 score of 84%, exceeding that of the average cardiologist with 78%. They tested their algorithm on the Challenge test dataset that was not used here and obtained a F1 score of 84% for AF detection. To note, this result was obtained after retraining their algorithm on the same 8528 records used in the current study. Our F1 score of 81%, without any previous retraining, shows a strong ability of the present AI platform to reach cardiologist-level accuracy, even with external datasets, which is an essential requirement in deploying deep-learning methods in clinical practice.

Although this is the first validation study of the AI platform on single-lead KardiaMobile records, the platform has already been used in other single-lead devices such as ICM. In a first study including 20 patients with 2261 records, performance metrics regarding detection of AF were as follows: 96.6% sensitivity, 95.4% specificity, 86.6% PPV, 98.9 NPV, and 91.3% F1 score.^13^ In a subsequent study investigating more cardiac-rhythm patterns through multilabel classification; performance metrics for AF with slow ventricular response; AF with controlled ventricular response; AF with rapid ventricular response were, respectively, 100%, 70%, and 88.6% for PPV, 66.7%, 65.6% and 98.5% for sensitivity, and 80%, 69.0% and 93.2% for F1 score.^12^ Together with the results of the current study, these published results suggest that the AI platform is robust and accurate for AF detection across different single-lead ECG devices such as ICM and handheld devices.

Previous evidence suggested that automated first-line ECG interpretation of AF was not accurate enough to support manual review, but current findings suggest that this statement may be revisited. Although previously published guidelines did not address the role of automated 1-lead ECG interpretation,^18^^,^^19^ a recent consensus statement suggests that abnormal ﬁndings obtained with this cutting-edge technology merit consultation with experts previously delivering a clinical decision.^20^ Our results align with this recommendation, enforcing the potential of automated 1-lead ECG interpretation for diagnosis of AF.

The recently released European Society of Cardiology (ESC) guidelines for the management of AF highlight the useful role of the novel ECG-based devices in AF first-line detection including those with 1 or 2 leads.^21^ In addition, these new guidelines emphasise the great impact AI is having in the electrophysiology automatic interpretation, while suggesting the current algorithms need to be further validated in external databases and real clinical settings. Therefore, our findings bring new evidence on the use of 1-lead ECG automatic analysis for the diagnosis of AF.

The SAFER (Screening for Atrial Fibrillation With ECG to Reduce Stroke) feasibility study assessed the level of agreement on detection of AF by independent cardiologists interpreting single-lead handheld ECGs.^22^ Their findings showed moderate agreement among cardiologists, indicating that in 70 of 100 screened participants, a different AF/non-AF diagnosis was delivered, depending on the expert who reviewed the ECG. In line with these results, another study from the VITAL-AF trial reported substantial variability in cardiologists’ interpretation of 1-lead ECGs.^23^ This evidence provides a strong rationale for aiming maximum reliability in the interpretation of single-lead ECGs. To that purpose, using an automated AI algorithm to detect AF is probably one of the most promising options in addition to cardiologist training on such ECG records.

Although the use of our Cloud-based and device agnostic platform to detect AF in 1-lead ECG records is encouraging, it should be emphasized that other arrhythmias and cardiac patterns could be detected by the embedded deep learning algorithms. As listed in Table 2, other cardiac patterns, such as different types of PVCs or PACs along with first-degree atrioventricular (AV) block were also detected using the present AI platform. Future research and expert labeling are needed to improve and fairly evaluate its performance to detect these additional cardiac patterns from 1-lead ECG records.

### Limitations

This study comes with some limitations. First, the used database originates from a public Challenge lacking clinical data and patient follow-up. As a result, demographic and clinical evolution data for the population are unavailable. This limitation prevented us from conducting meaningful association analyses between the obtained metrics and patient characteristics. Subgroup analysis could have been performed to compare performance metrics in patients with and without symptoms. To note, an ongoing clinical trial, the WILLEM trial (NCT05890716), has been designed to evaluate the performance of the AI platform in different centers, countries, cardiac diseases, and ECG machines, providing demographic and clinical data that will enable such subgroup analysis and description of patient characteristics. Second, expert cardiologists have contributed to establish the gold standard based on 1-lead ECG interpretation. Therefore, given the observed variability between cardiologists in AF diagnosis from 1-lead records,^22^^,^^23^ and in the absence of 12-lead records and clinical follow-up, some cases might have been wrongly classified as FP or FN. However, the thorough labeling process used has probably minimized those misclassifications. Validating AI interpretation of 1-lead ECG record with 12-lead cardiologist interpretation as ground truth would be an interesting perspective. Third, as the ECG data analysed were recorded with the KardiaMobile handheld device, generalizations of our results must be done with caution. The AI platform capabilities should be also validated with 1-lead ECG records coming from other mobile devices as smartwatches. Finally, the manual revision of false positive and false negative cases has mainly revealed that AI mistakes occurred in presence of artefacts such as baseline oscillations and high-frequency noise (Figure 2). Future work might include new signal processing techniques to either automatically identify artefacted records in which the AI platform should not be used or to correct or filter those records before further cardiac pattern identification.

### Strengths

Our AI platform was not previously trained with the present AF dataset Challenge and depicts a true external validation setting. The large sample size used for AI validation in this study demonstrates the high performance of the current algorithms. The labeling process quality was high, considering the initial iterating process used during the Challenge and the added expert review. The Cloud-based platform has already obtained regulatory clearance in Europe; therefore, it could be promptly applied for AF diagnosis support in real clinical settings.

## Conclusion

We provide the external validation of the existing Willem AI platform to detect AF from 1-lead ECG records. Its cardiologist-level detection performance could facilitate remote AF screening and supports cardiologists’ AF diagnoses, reducing bias and standardizing the manual review process.

Whether the high accuracy shown with 1-lead ECGs could enable to replace the standard 12-lead ECG interpretation requires further validation with prospective data and patient follow-up. However, our results already suggest screening strategies using 1-lead ECG recorders coupled with Cloud-based AI interpretation could help cardiologists to detect and treat AF early. Interestingly, other cardiac arrhythmias may be also detected from 1-lead records by such deep-learning platforms, but this insight needs further investigation and validation.

## Disclosures

Dr Lillo-Castellano and Dr Marina-Breysse are founding members of IDOVEN. Drs De Guio, Lizcano, Corrochano-Diego, Jimenez-Virumbrales, and Toribio-Fernandez are employees and part of the AI research team of IDOVEN. Dr Rienstra has received support from an unrestricted research grant from the Dutch Heart Foundation, which is conducted in collaboration with and supported by the Dutch CardioVascular Alliance, 01-002-2022-0118 EmbRACE. He has received an unrestricted research grant from ZonMW and the Dutch Heart Foundation; DECISION project 848090001; unrestricted research grants from the Netherlands Cardiovascular Research Initiative: an initiative with support of the Dutch Heart Foundation: RACE V (CVON 2014–9), RED-CVD (CVON2017-11); unrestricted research grant from Top Sector Life Sciences & Health to the Dutch Heart Foundation (PPP Allowance; CVON-AI (2018B017)); unrestricted research grant from the European Union’s Horizon 2020 research and innovation program under grant agreement: EHRA-PATHS (945260); consultancy fees from Bayer (OCEANIC-AF national PI), and InCarda Therapeutics (RESTORE-SR national PI) to the institution.
